# Tapping Into the Language of Touch: Using Non-invasive Stimulation to Specify Tactile Afferent Firing Patterns

**DOI:** 10.3389/fnins.2020.00500

**Published:** 2020-05-19

**Authors:** Richard M. Vickery, Kevin K. W. Ng, Jason R. Potas, Mohit N. Shivdasani, Sarah McIntyre, Saad S. Nagi, Ingvars Birznieks

**Affiliations:** ^1^School of Medical Sciences, UNSW Sydney, Sydney, NSW, Australia; ^2^Neuroscience Research Australia, Sydney, NSW, Australia; ^3^Graduate School of Biomedical Engineering, UNSW Sydney, Sydney, NSW, Australia; ^4^Center for Social and Affective Neuroscience, Department of Biomedical and Clinical Sciences, Linköping University, Linköping, Sweden

**Keywords:** bionic, tactile, neural prosthesis, brain-machine interface, somatosensory, spike train, rate code, neural coding

## Abstract

The temporal pattern of action potentials can convey rich information in a variety of sensory systems. We describe a new non-invasive technique that enables precise, reliable generation of action potential patterns in tactile peripheral afferent neurons by brief taps on the skin. Using this technique, we demonstrate sophisticated coding of temporal information in the somatosensory system, that shows that perceived vibration frequency is not encoded in peripheral afferents as was expected by either their firing rate or the underlying periodicity of the stimulus. Instead, a burst gap or silent gap between trains of action potentials conveys frequency information. This opens the possibility of new encoding strategies that could be deployed to convey sensory information using mechanical or electrical stimulation in neural prostheses and brain-machine interfaces, and may extend to senses beyond artificial encoding of aspects of touch. We argue that a focus on appropriate use of effective temporal coding offers more prospects for rapid improvement in the function of these interfaces than attempts to scale-up existing devices.

## Introduction

A sensory brain-machine interface bypasses the default systems of sensory input that transduce environmental signals into neural activity. Instead, neural activity is generated in new ways, driven by computer inputs that are developed based on environmental signals. Improved sensory brain-machine interfaces offer promise in many fields, from quality of life for those with a disability, to augmenting the range of normal senses. One of the major challenges of sensory brain-machine interfaces has traditionally been viewed as the issue of spatial and numerical scale: for example in humans, the optic nerve has the order of 10^6^ axons (Mikelberg et al., 1989), the auditory component of the vestibular cochlear nerve has the order of 10^4^ axons (Spoendlin and Schrott, 1990), and the median nerve arising from the hand also has the order of 10^4^ axons (Johansson and Vallbo, 1979). In contrast, current retinal implants have 20 to a few hundred electrodes (Sinclair et al., 2016; Farvardin et al., 2018), the cochlear implant that stimulates surviving spiral ganglion cells has less than 25 electrodes (Patrick and Clark, 1991), and bionic hand prostheses that aim to provide user feedback as touch sensations use implants with 4 to 96 electrodes on up to three nerves (Boretius et al., 2010; Raspopovic et al., 2014; Schiefer et al., 2016; Wendelken et al., 2017). Considerable effort is being expended to bridge the gap between the number of sensors/electrodes and the number of afferent neurons. In this review we hope to provoke reflection on the tractability of this challenge and draw attention to the potential offered by a closer focus on precise control of the timing of inputs through these interfaces. In contrast to the spatial challenges, a sub-millisecond time resolution is easily achieved by any of the current interfaces, and is directly comparable to the time scale of the nervous system, which uses action potentials or “spikes,” with a duration of around one millisecond. The language of the brain is spoken in the temporal pattern of these spikes, as well as the array of neurons in which they are active. More focus on this temporal patterning may represent a tractable parallel path to advance the quality of sensory neural prostheses. We will have a particular focus on recent findings in the tactile system, and their implication for efficient encoding of information for relay to the brain.

## The Nature of Neural Information

The last 100 years have revealed an unprecedented amount about the workings of the nervous system. It is now well understood how voltage-gated ion channels support the transmission of all-or-nothing action potentials in a reliable, rapid manner over long distances (Hodgkin and Huxley, 1952). The conversion of this action potential into a pulse of neurotransmitters that engage with receptors on post-synaptic elements at the synapse is also largely understood (Lisman et al., 2007). What has lagged behind is our understanding of the information content of these events. At a certain point in the neural processing of a sensory event, the entire information content of the event has to be conveyed in the pattern of action potentials travelling in the axons of afferent neurons. Each of the action potentials is just a brief alteration of the membrane potential, but somehow these flickering potentials can convey essential qualities of an event, such as the warmth, texture, shape and firmness of a hand that one is holding.

Some of the information is inherent in the nature of the afferent neuron and the environmental signals it is able to transduce. For instance, cold sensitive afferents express TRP channels in their cell membrane that open when cooled, leading to depolarisation and generation of action potentials (Bautista et al., 2007). Action potentials in these axons signal “cold” because that is the most common origin of action potentials in these axons, and because they are connected to other neurons higher in the nervous system that take part in cold-related behaviours such as shivering. A single action potential in these axons has this property of “cold,” even if it is elicited in the axon by something other than the opening of TRP channels such as electrical stimulation.

However, the more detailed information about timing, intensity, and complex stimulus properties, are conveyed by the pattern of firing of multiple action potentials in each axon. The default level of neuroscientific analysis of these firing patterns has been to convert them to a mean rate for use as an index of intensity of afferent activation. This is a simple and robust approach, but ignores a long history of research into the role of temporal encoding in auditory system (Galambos and Davis, 1943) and considerable evidence of a potential role for action potential timing in a variety of sensory systems (VanRullen et al., 2005). A rate-based approach is also used as the default encoding strategy of many sensory prostheses, in part because these devices simultaneously activate large populations of afferents, and because of the view that the temporal information will be recoded to a rate code anyway at higher levels of the nervous system (Ahissar, 1998). This rate-based approach discards the temporal relation between individual action potentials which we will show is potentially a rich source of information.

## Neural Information in Touch

Touch is an excellent sensory system in which to explore questions of neural information encoding for a number of important conceptual and practical reasons. There is a considerable body of existing research that suggests that the tactile system may encode information in multiple different ways, some of which depend on precise temporal features of action potential patterns. The tactile nervous system transduces a rich and varied set of stimuli that convey critical information for often subconscious manipulation, but also contributes to conscious and affective experiences. The afferent axons are generally readily accessible for invasive and non-invasive stimulation and recording in the tactile system. This property makes the system an excellent site for a sensory neural prosthesis, as evidenced by the development of increasingly sophisticated closed-loop technologies for prosthetic limbs and haptic devices.

The sense of touch at the fingertips is subserved in humans by four classes of myelinated afferent neurons, reviewed by Johnson (2001), Macefield and Birznieks (2009), and Abraira and Ginty (2013). Some of these afferents, called Fast Adapting (FA), respond only to dynamic stimuli that induce a time-varying strain profile across their receptor endings. Others, called Slowly Adapting (SA) also respond to dynamic stimuli, but are able to produce a response sustained over many seconds to static stimuli. The hand contains approximately 17 000 of these afferents (Johansson and Vallbo, 1979), and their combined activity is sufficient for us to discriminate shapes, textures, contact forces, vibration frequencies and directions of movement. Long-standing research using vibration of punctate probes on the skin has established a set of frequency sensitivity profiles for the four fast touch afferent types found on the hand (Talbot et al., 1968; Johansson et al., 1982). However, the extent to which the information from these different afferent types is maintained in separate channels, and how information in the firing pattern of action potentials conveys the sinusoidal stimulation frequency and the stimulation amplitude remains an area of active enquiry.

The four different afferent classes have their peak sensitivities at different sinusoidal vibration frequencies. For SAI and SAII afferents, their best vibration sensitivity is at low frequencies, below 8 Hz, while FAI are most sensitive at 32 Hz, and FAII at 256 Hz (Johansson et al., 1982). Even though individual afferents will respond to a wide range of frequencies given a strong enough stimulus (Johansson et al., 1982), a prominent interpretation of the frequency sensitivity profiles of these afferents is that, similar to the “cold” property of a cold afferent, the four mechanosensitive afferent types (SAI, SAII, FAI, and FAII) each give rise to a qualitatively different sensation of frequency. The most developed of these interpretations is the four-channel model of mechanoreception which assigns each afferent type to part of the frequency range based on behaviourally-determined thresholds (Bolanowski et al., 1988). In this model, the SAII are assigned to a high frequency range, and it is suggested that channels interact by summation of their perceived magnitudes (Gescheider et al., 2004). However, this interpretation makes a logical leap that links the frequency-dependent thresholds of individual afferent types, and frequency-dependent variation in perceptual thresholds, to conclude that single afferent types directly and independently mediate the perception of frequency in particular frequency bands.

An extension of this interpretation addresses how to reconcile the signals when multiple afferent types are simultaneously active. In this case, it was hypothesised that the ratio of their activities could encode vibration frequency in a manner analogous to colour-sensitive cone cells in the retina, with the most active afferent types contributing most to frequency perception. However, a study that systematically varied the recruitment ratios of the FA afferents failed to show any consistent effect on perceptual judgements of frequency (Morley and Rowe, 1990). Indeed, more recent evidence from animal studies show that the signals deriving from these afferents converge at the primary somatosensory cortex, or perhaps even lower levels (Pei et al., 2009; Carter et al., 2014; Saal et al., 2015). This suggests that we should not treat these afferents as pure channels representing a frequency band, and supports the idea that the information from these channels is integrated in a way that is somewhat agnostic about the afferent source.

**FIGURE 1:**
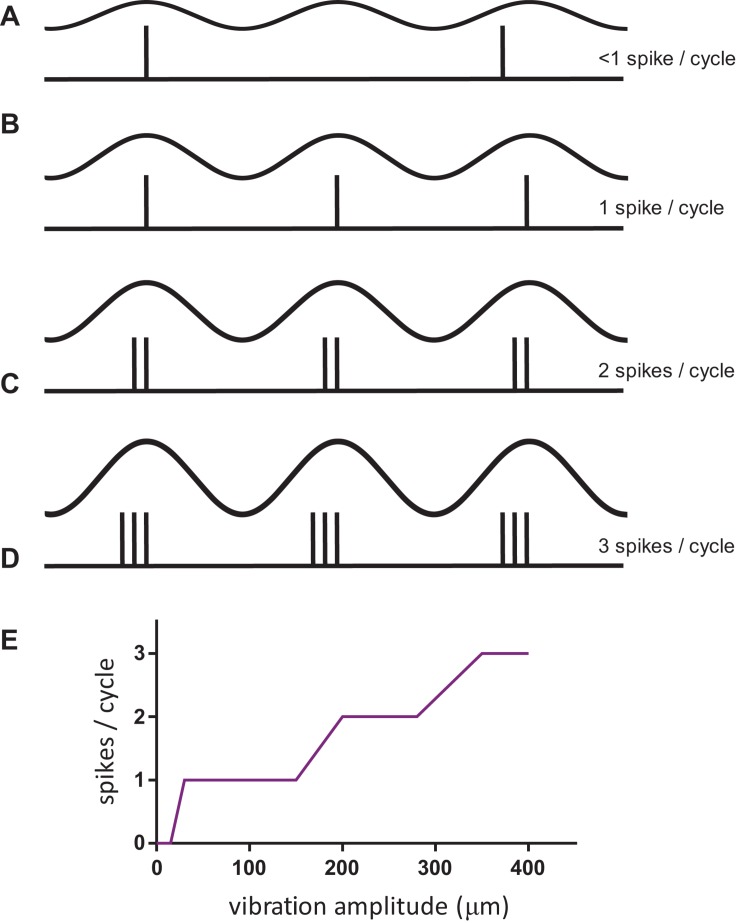
The complex relationship of afferent response with stimulus amplitude for sinusoidal vibration on the skin. **(A–D)** The top trace in each condition represents the stimulus, in the bottom trace a vertical inflection represents an action potential or spike in one afferent neuron. **(E)** Schematic of FAI afferent response when stimulated at 50 Hz over a range of vibration amplitudes. Notice the plateaus in the response over a range of vibration amplitudes, in particular at 1:1 entrainment.

The question of the neural code for frequency, and how to extract it independently of the stimulus amplitude, is challenging to answer as shown in Figure 1. At low amplitudes of stimulation (but above the threshold for a neural response), the tactile afferent neuron will generate an occasional action potential during a cycle of vibration that moves the probe down and up on the skin. At a higher amplitude, the afferent will generate 1 spike for each vibration cycle, and the response rate will match the stimulus frequency; this response pattern is termed 1:1 entrainment or the tuning plateau (Talbot et al., 1968). At even greater amplitudes, the afferent may respond 2, 3, or more times for each cycle of vibration (Johnson, 1974; Johansson et al., 1982). This relationship of amplitude and frequency implies that a neural code based on counting the number of action potentials in a fixed time period (rate code) could possibly be used to determine the amplitude (Figure 1E) but cannot be used to determine the frequency of the stimulus.

## Evidence for the Importance of Timing Information in Sensory Neural Signals

Mountcastle’s group advanced arguments for a neural code based on the periodicity of inter-spike intervals in neural firing as a means by which the nervous system could distinguish frequency from intensity in a vibratory stimulus (Talbot et al., 1968). This is a time-based code operating on the pattern of spikes to signal frequency, which could function independently of the underlying response rate of the neuron. No specific neural mechanism was proposed to perform this decoding, which was envisaged to operate in part by comparison across cortical neurons, as individual neurons cannot fire at frequencies matching the entrainment rate. Even 50 years later, no easily interpretable neural code or decoding mechanism for vibrotactile frequency in cortical neurons has been discovered, other than an apparent special case of place coding in mice inputs related to deep Pacinian inputs (Prsa et al., 2019).

The neural code for the intensity of the vibratory stimulus was proposed by Johnson (1974) and later Muniak et al. (2007) to be based on either the number of active afferents, or a code based on the firing rate in these afferents.

Over the last 50 years, there has been increasing evidence to support an important role for timing information such as that based on phase-locking proposed by Talbot et al. (1968) rather than simply relying on the spike rate in sensory inputs. This has been demonstrated for hearing using both normal audition (von Békésy, 1961) and by stimulation through a cochlear implant, using pulse trains that alternated 2 intervals (Carlyon et al., 2008), where the perceptual frequency was not that expected from the simple arithmetic rate of pulses presented. There is suggestive evidence from visual experiments in animals (Gollisch and Meister, 2008) where it was shown that just the latencies of the very first spike from multiple retinal ganglion cells to a flashed visual image were sufficient to enable reconstruction of the image. The results were even more robust when latency differences between neurons were used, and other similar studies are reviewed in VanRullen et al. (2005) and Panzeri et al. (2014). For these measurements, stimulus onset, which in nature would be triggered by a rapid eye movement, acts as the base time point from which latencies are measured. There is similarly suggestive data for the significance of equivalent first spike timing in the tactile discrimination of force direction and object shape (Johansson and Birznieks, 2004), torque (Birznieks et al., 2010; Redmond et al., 2010), contact force and friction (Khamis et al., 2014). Analyses of inputs from FAI and SAI afferents suggest that fine-grained temporal information can be used to improve discrimination of the edge orientation of tactile objects (Pruszynski and Johansson, 2014).

Animal studies with tactile stimuli also indicate that temporal codes are important (Panzeri et al., 2001; Arabzadeh et al., 2006). In experiments in awake behaving rats that made texture judgements using their whiskers, it was shown that time-based measures carried greater information (Zuo et al., 2015). The time-based measures were created by determining a template that weighted spike contributions, based on their time after whisker contact, to produce best discrimination. A challenge in many such studies is that demonstrating that timing conveys more information than rate alone, is not the same as showing that the nervous system makes use of this available information. This study by Zuo et al. (2015) begins to bridge the gap between what spike timing information, in particular relative spike timing information from an ensemble neural population, might enable, and what it is actually used for, by showing that these timing measures were better predictors of the actual correct-incorrect decision that the animal made about the texture than rate-based measures. This suggests that although the exact timing mechanism employed by the experimenters is unlikely to be what is implemented in the nervous system, the nervous system is actually employing some form of analysis of time-based information. In a study on discrimination between different fabrics on a rotating drum, human behavioural performance was compared with possible temporal and rate codes based on recordings in monkeys from single tactile afferents innervating the fingers (Weber et al., 2013). The evidence was clear that judgements about fine textures were predominantly based on temporally-coded information arriving via FA afferents, whereas coarse textures depended on a population rate code in SAI afferents.

## Control of Tactile Afferent Spike Timing by Non-Invasive Stimulation

The two studies described above were able to unite stimulus control, neuronal recording and behavioural experiments in awake behaving animals, but obtaining equivalent data in humans is particularly challenging. In our laboratory, we have been able to unite two technologies and bring a new approach toward trying to resolve these questions of information transmission in the peripheral nervous system for touch. One technology is pulsatile stimulation, which offers a non-invasive way to induce precise patterns of single action potentials in a small population of peripheral afferent neurons. The other technology is microneurography (Vallbo and Hagbarth, 1968), which enables us to record activity in real time from single afferent neurons in awake humans. The combination of these techniques with psychophysics enables us to confidently interrogate questions of the neural coding of complex tactile properties, by giving us near complete control over the ascending afferent activity patterns (Birznieks and Vickery, 2017).

**FIGURE 2:**
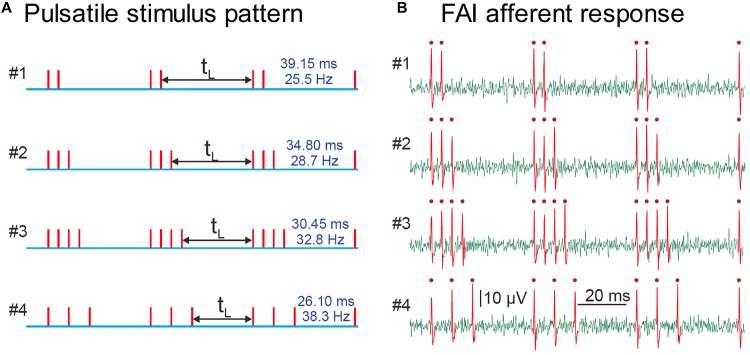
**(A)** Representation of four pulsatile vibrotactile stimuli, where each vertical line indicates the time of the mechanical pulse. **(B)** Recording of spike trains evoked in a single human FAI tactile afferent, identified single spikes are shown in red with a dot. There is a precise match of stimulus and afferent response, with one spike per pulse. Adapted with permission from Birznieks and Vickery, Spike Timing Matters in Novel Neuronal Code Involved in Vibrotactile Frequency Perception, *Current Biology*, 27, 1485-1490, 2017, Elsevier Ltd., doi.org/10.1016/j.cub.2017.04.011.

The stimulation technique relies on creating precisely-controlled spike patterns in tactile afferents by using brief taps (stimulus pulses) delivered at intensities well above the neural response threshold. Provided the duration of the pulse stimulus is approximately the same as the refractory period of the afferent axon (around 1.5 ms under normal conditions), each pulse will induce a maximum of a single spike in responding afferent axons over a wide range of stimulus amplitudes. This technique enables us to reproduce a desired spiking pattern in human peripheral afferent axons, and perform psychophysical experiments to interrogate the sensation elicited. The pulses can be repeated at any desired timing and repetition rate, while always activating the same population of afferent neurons. In this way, we can simulate varied environmental parameters by creating spiking patterns that reflect those that the environmental condition would have elicited, but maintain a fixed afferent population that drives the sensation. We use the technique of microneurography to validate the fidelity of the conversion of our stimulus into spike patterns by recording from single human tactile afferents while the subject receives the pulsatile stimulation (see Figure 2).

The pulsatile stimuli used in our experiments are all delivered non-invasively, either by mechanical stimulation of the skin, or by electrical stimulation of the skin overlying a peripheral nerve such as the digital nerve. For electrical stimulation, we use an isolated stimulator such as the DS5 (Digitimer, Hertfordshire, United Kingdom) to deliver charge-balanced stimulation, with a depolarising phase of 0.1 ms, and a repolarising phase of 1 ms. For mechanical stimulation we have several devices capable of producing a brief mechanical pulse. We have used an Optacon 1D (Bliss, 1969) driven by a custom-built interface which offers 144 pins with approximately 15 μm displacement over <2 ms. The amplitude and pulse width are adequate to reliably activate FAI and FAII afferents, but not SA afferents, which is consistent with findings in the monkey (Gardner and Palmer, 1989). To obtain larger pulse amplitudes that recruit SA afferents, we use a GW-V4 shaker (Data Physics, San Jose, United States) controlled via a Power1401 (CED, Milton, United Kingdom) where we use feedforward control to damp the resonance of the shaker to ensure a brief single mechanical pulse at amplitudes up to 150 μm.

The use of low amplitude (<5 μm) pulsatile stimulation enables selective activation of FAII afferents by exploiting their extreme sensitivity to short-period waveforms (Johansson et al., 1982). At frequencies below 40 Hz, tactile inputs are normally dominated by FAI afferents because they are activated at a lower threshold amplitude than FAII afferents when sinusoidal vibration is used, as has been the case in most experiments using vibrotactile stimuli. However, by using our low amplitude pulsatile stimuli, we are able to activate FAII afferents selectively without FAI activity, even at low frequencies. These non-invasive stimulation tools enabled us to demonstrate that the FAII afferents are capable of sustaining high-quality vibration perception at these low frequencies, which provides further support for the convergence of these input channels onto common cortical frequency processing circuits (Birznieks et al., 2019).

## Modifying Human Touch Sensation by Varying Spike Timing Patterns

**FIGURE 3:**
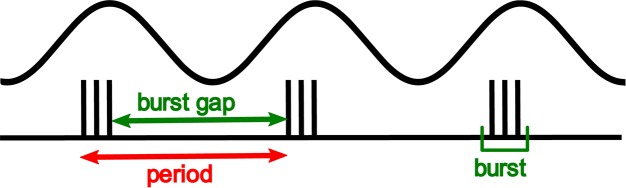
Shows the grouping of spikes into bursts. The apparent frequency is the reciprocal of the time between bursts, labeled the burst gap. This is higher than the frequency predicted from the period of the base stimulus waveform.

Through the use of these non-invasive stimulation techniques, we have been able to demonstrate the critical importance of spike timing in shaping human tactile perception, in this case, of vibrotactile frequency. We set out to show that the spike rate in peripheral afferents could not plausibly code for vibration frequency, informed by the intuition from Figure 1, that increasing vibration amplitude leads to more spikes per cycle, but does not produce an equivalent upward shift in the perceived frequency. Using controlled pulsatile stimuli such as those shown in Figure 2, which are a controlled way of simulating the burst firing illustrated in Figure 1, we demonstrated that the perceived frequency was not related to the spike rate (Birznieks and Vickery, 2017). Unexpectedly, however, we were not able to demonstrate that the perceived frequency was related to the underlying periodicity of these stimuli in accordance with the hypotheses of Talbot et al. (1968). Instead, we found that subjects’ perceived frequency was best explained by the silent gap between the bursts of spikes irrespective of the number of spikes within a burst, burst duration or periodicity. The difference between the burst gap and the periodicity is illustrated in Figure 3. Bursts are well-known in the neuroscientific literature as a common form of neural activity in the tactile system (Vickery et al., 1994) and elsewhere. Bursts have been speculated to play a key role in information processing by providing precise information that can be reliably signalled across the next relay (Lisman, 1997), by interacting with resonant frequency tuning of relay cells (Izhikevich et al., 2003) and by offering a parallel information path in the form of bursts contrasted to isolated spikes (Naud and Sprekeler, 2018). In these three studies, the definition of a burst’s duration ranged from 10 to 25 ms, with this difference likely dependent on the temporal integration properties of the neurons studied. We determined the time envelope within which subsequent spikes in our study would be grouped together as a burst, by determining the range of pulse separations over which the burst gap applied. We found that spikes in the tactile afferents with a 15 ms envelope were treated as a burst, and over the range of 15–25 ms, there was still some interaction. Beyond 25 ms the spikes were treated as independent sensory events and the burst gap code no longer applied and perception could be explained by a rate code (Birznieks and Vickery, 2017). We have now extended these findings to show that we can elicit the same burst gap responses when we deliver transcutaneous electrical stimulation to digital nerves instead of using mechanical stimulation (Ng et al., 2020).

To demonstrate the robustness of the integration envelope for spiking patterns containing bursts, we have also tested aperiodic stimuli (Ng et al., 2018) that may better model the variation encountered in day-to-day tactile exploration of surfaces. Using the Optacon, we delivered spike patterns with intervals ranging from 4 to 113 ms with mean spike rates below 50 Hz. Our prediction was that the perceived frequency of these stimuli would be lower than the mean spike rate as a result of the intervals of <25 ms falling within the burst window and so not contributing their weights to the apparent frequency. All three frequencies tested showed perceived frequencies approximately 80% of that which would be expected from the mean rate (Ng et al., 2018). This provides compelling evidence that mean spike rate is not the key determinant of perceived frequency, and that the fine temporal structure of spike trains plays a critical role in the sensory experience. This is consistent with experimental results that pooled afferent data from monkeys with psychophysics studies conducted in humans to show that fine temporal features on a millisecond scale detected by FA afferents were far stronger predictors of human perception of the similarity of two stimuli than were measures based on a rate code (Mackevicius et al., 2012).

## Implications for Design of Sensory Neural Prostheses and Brain-Machine Interfaces

The insights into neural coding, and the opportunity afforded by control of spike timing, represent advances that should be translatable into advances in interfaces between external devices and peripheral nerves or neurons in the central nervous system. Although current bionic prostheses offer a profound benefit for users, as outlined in the introduction, they are nowhere near matching the scale of the sensory transduction systems that they are designed to interface with. Any currently foreseeable improvement would still leave interfaces a long way from a 1:1 connection between sensor and afferent neuron. This challenge has several dimensions, one is to maintain a stable connection with a neuron or axon that is fragile, flexible, and has a size on the order of 10 μm. Although there are possible new approaches using flexible materials and optical technologies, there remain very significant challenges to be overcome (Durand et al., 2014).

Assuming the problem of scale can somehow be overcome by increasingly miniaturised technology, the 1:1 sensor-afferent relationship can only work efficiently if the afferents can be mapped so that it is known what sensation each afferent gives rise to. This enables computational processing of the sensor signals to optimise the sensory experience by stimulating each afferent with input from appropriate sensors in appropriate patterns. For some afferents, this mapping should prove straight-forward, as it seems that in general single FAI and FAII afferents can give rise to a clear and localised percept (Ochoa and Torebjörk, 1983; Vallbo and Johansson, 1984) and SAI from the dorsum of the foot and hand can evoke conscious sensation (Nagi et al., 2019). In contrast, single SAII afferents (Ochoa and Torebjörk, 1983), and SAI afferents from the hairy skin of the forearm (Vickery et al., 1993), do not appear to give rise to a conscious percept. It may be that activation of certain combinations of these afferents can evoke a perception but the complexity and dimensionality of these combinations are currently not understood.

Without a 1:1 scale, the bionic prosthesis is left to activate groups of neurons rather than single afferents. In a relatively homogeneous sensory system such as the auditory system which has only inner and outer hair cells as the major neural distinctions in the cochlea, this strategy may prove effective. In vision, the photoreceptors converge in complex ways onto ganglion cells, creating, among others, on-centre and off-centre pathways. Simultaneously stimulating groups of on-centre and off-centre cells creates an unnatural perception, as normally only one or the other type would be active for a particular retinal location at any instant, and this may partly explain why retinal stimulation produces phosphenes (Sinclair et al., 2016) rather than more natural percepts. In the tactile system, a bionic prosthesis activating groups of afferents will likely activate both slowly adapting and rapidly adapting neurons, thereby limiting the ability to tailor the stimulation strategy to suit the particular afferent type. Using mechanical stimulation instead of electrical stimulation enables some selectivity over which tactile afferent types are recruited (Antfolk et al., 2013; Birznieks et al., 2019).

An important related question is the extent to which a single afferent should be treated as a unique individual input, rather than as a representative of an afferent class. The type of nerve ending and its distance from the stimulus site matters, but every tactile afferent has its own unique sensitivity profile whereby it is more efficiently excited than other afferents by certain features of a given stimulus due to variations in receptor embedding in skin tissue, such as geometry and anchoring (Birznieks et al., 2001). Natural stimuli will likely activate afferents of all classes (Johansson and Westling, 1987), however, any given afferent may contribute to perception in a very specific way in one situation but not at all in another situation. It is currently an open question as to how much the higher levels of the nervous system take advantage of these highly specific sources of information. Presumably, correlation of stimulus and particular individual afferent activations could be learned over the course of development through neural plasticity to inform decision making. A prosthetic replacement stimulus ideally would harness the same neural plasticity to maximise the information that can be conveyed.

The semi-selective mechanical stimulation proposed above could be combined with targeted sensory reinnervation surgery in amputees, where the stumps of the nerves that would normally have innervated the hand (ulnar and median) are surgically repositioned and stimulated to grow, so that they innervate the overlying skin of the new site which has been de-innervated. Mechanical stimulation of the skin at this new site would be able to provide a more natural mapping of sensation from different afferent types to the bionic hand, as the sensation would appear to the subject to arise from their hand, rather than from the body site actually stimulated (Hebert et al., 2014).

## Using Temporal Neural Codes to Improve Sensory Neural Prostheses

We suggest that a renewed focus on understanding, and deploying, precise temporal information in the induced spike patterns can help realise better outcomes for bionic prostheses. Unlike the problem of spatial and numerical scale, the timescale of the nervous system is very tractable with current technology. A time resolution of 0.2 ms, translating to a digital to analog conversion (DAC) rate of 5 kHz per channel, is almost certainly sufficient to capture the full temporal resolution of the nervous system (Mackevicius et al., 2012) except in the auditory domain for sound localisation by inter-aural time differences where thresholds can be 0.01 ms (Brughera et al., 2013). There are two approaches to better encode this temporal information, which vary in the extent of information interpretation required.

One strategy is to take an agnostic view of the salience of the patterns, and instead simply relay the temporal information as realistic spiking patterns as faithfully as possible. The strength of such an approach is that potentially useful information, whose encoding we do not yet understand, is not discarded. This approach is behind one technique we have employed (Rager et al., 2013) to preserve spike firing patterns related to environmental features down to sub-millisecond precision. We built a library of virtual tactile afferent neurons by training noisy integrate-and-fire neurons (Paninski et al., 2004) on data derived from real afferents while driving them through artificial sensors given the same set of mechanical stimuli. We accepted that we would sacrifice spatial scale by using only a few transducers in place of the thousands of normal tactile receptors, but we were able to preserve spike firing patterns at high temporal resolution. A related approach, based on TouchSim which models afferent population responses (Saal et al., 2017), uses pooled outputs from the model (implemented in an efficient coding algorithm) to capture both spiking patterns and number of active afferents (Okorokova et al., 2018). This approach performs well and shows good modulation with variations in stimulus intensity, but may lose some time fidelity through pooling which is apparent at frequencies above 60 Hz (Okorokova et al., 2018).

The other strategy is to try and determine how sensory information is conveyed in the temporal patterning of spike firing. This approach enables synthesis of desired sensation by creating the appropriate spike pattern, as well as advances our basic neuroscientific understanding. However, as the progress of more than 50 years of research outlined above shows, there remains much to be learned. The insights about how frequency is encoded by the burst gap rather than the period represent one small step in this direction. We are currently exploring whether the spikes that are “hidden” inside the burst envelope in peripheral afferent spike patterns may contribute to other aspects of tactile sensation such as intensity (Ng et al., 2019). Other groups are combining animal experiments with single unit cortical recording and behavioural experiments to understand the neural code for tactile information at higher levels of the nervous system (Harvey et al., 2013). Studies in the barrel cortex of mice, an important tactile area for whisker inputs in rodents, suggests that strong integration of whisker movements occurs over a short time period of less than 25 ms (Stüttgen and Schwarz, 2010; Estebanez et al., 2012; Tsytsarev et al., 2016), which fits well with our observations. Weaker integration of one or two inter-spike intervals over longer time periods has also been reported (Pitas et al., 2016), which supports the importance of temporal pattern encoding, and may underlie the recognition of the burst gap intervals.

**FIGURE 4:**
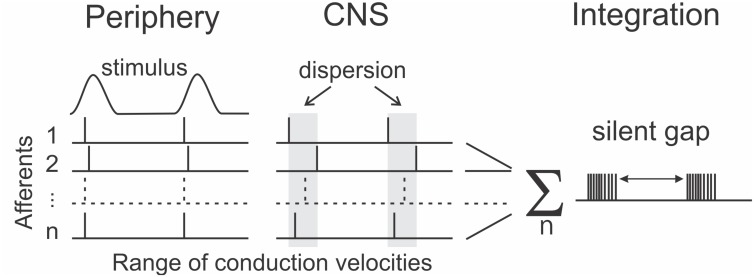
Extending the concept of the burst gap for encoding frequency to span the spiking activity across a population of afferents (1…n) as the “silent gap.” The near simultaneous events in the periphery become dispersed in time on arrival at the central nervous system (CNS) due to differences between afferent conduction velocities.

An open question is whether it is sufficient to examine temporal coding in spike patterns of single afferents or whether temporal patterning should be considered as extending across a population of afferent neurons. One challenge facing a population-based model is the dispersion in arrival times at the central nervous system (CNS), of spikes travelling in different afferents that originate from a single tactile event in the periphery. The conduction velocity of human afferent axons varies from 35 to 70 ms^–1^ between axons in a single nerve (Dorfman, 1984). Over the approximately 1 m conduction distance from fingertip to brainstem, this velocity difference translates to a difference in arrival times of about 15 ms. This is a close match to the time envelope defining a burst that we discovered for tactile afferents, and opens the possibility that one aspect of burst gap encoding is to preserve unity of sensation arising from the spikes produced by a single peripheral event. By ignoring the scattered spikes, the nervous system can reliably distinguish a single event. This suggests a modified form of the burst gap, which we have termed the “silent gap,” where the burst is defined by spiking activity aggregated across afferents as shown in Figure 4. This aggregation would occur at the first central nervous system synapse, which for the main tactile pathways are in the dorsal column nuclei. These nuclei are also a focus for a possible brain-machine interface, with early work showing potential for decoding the afferent input signals using machine-learning techniques (Sritharan et al., 2016; Loutit et al., 2017, 2019; Loutit and Potas, 2020).

## Conclusion

Although it is clear that there is still far more to be understood about the information encoded by the temporal patterning of spikes, it is also clear that this represents a relatively under-utilised tool to improve sensory neural prostheses and brain-machine interfaces. The tractability of precisely controlling temporal features, when compared with the many challenges of other ways of improving these interfaces, suggest that basic neuroscientific research needs to continue to advance the field, but that current understandings should be built into the next generation of devices. It is likely that tactile and auditory prostheses will show the most benefit from the introduction of temporal-based encoding as the use of time-based information is best understood in these sensory systems; but further investigations will likely reveal ways to deploy this usefully in other modalities.

## Author Contributions

RV wrote the first draft of the manuscript. All authors contributed to conception of the project, manuscript revision, and read and approved the submitted version.

## Conflict of Interest

The authors declare that the research was conducted in the absence of any commercial or financial relationships that could be construed as a potential conflict of interest.
